# Rheumatism

**Published:** 1845-06

**Authors:** 


*Rheumatism.*—The prognosis is generally favorable so far as life is concerned, for it is a fact that no fever is so seldom fatal as rheumatic fever; but how long the disease will contiuue, or how frequently the patient may relapse, it is impossible to predict. It is one of the most painful and vexatious inflictions to which humanity is subjected. When several joints and the muscles moving them are simultaneously affected, the sufferings are very severe and the pains excruciating. The sufferer cannot bear to be moved, or scarcely touched. Even in its subdued form it is still very painful, and is not easily or patiently endured. The physicians of England tell us it often terminates in fatal disease of the heart; I have not been so unfortunate as to meet with such a case. I have known the same patient suffer nine or ten attacks in the course of twenty years, and survive them all and enjoy good health. The convalescence is frequently tedious and protracted: sometimes, however, the cure is rapid and permanent.

There have been various plans of treatment proposed and followed, such as the depleting, the narcotic, the tonic, and the stimulating; of which I shall now take some notice and briefly indicate my preferences.

Blood-letting is always the first remedy suggested. When carried to an extent sufficient to subdue the disease, it leaves the patient in a state of great prostration, and dooms him to a slow convalescence. It seems to have more control over the accompanying fever than over the local pain and swelling. On the whole, I am not in favor of excessive bleeding in rheumatism, & in many cases never use the lancet at all. I choose to commence the treatment by giving a full dose of calomel, say fifteen grains, followed byinfusion of senna, or a dose of magnesia and rhubarb: then I give tartar emetic in small doses, and warm drinks to promote diaphoresis, and a pill of opium, two grains, or a teaspoonful of laudanum at night. Opiates I consider indispensable from the commencement to the termination of the disease. The employment of calomel and opium in small doses I have seldom found to have any beneficial effects. Thus exhibited, calomel is tolerated, rheumatic patients not being easily salivated: but the disease is not relieved. Opium in substance, or laudanum or the salts of morphine, in full doses, are always useful. To give temporary ease and to procure sleep are very desirable, and this composure tends to the resolution of the disease. This is all I have to say concerning the depleting and the soothing plans of treatment.

Respecting the tonic plan so warmly advocated by Dr. Haygarth in the last century, and lately revived in England, and in this country, I have no favorable report to make. The quinine has always aggravated the pain, and rendered the pulse fuller and harder. The late Dr. Bushe told me he had cured a case of acute rheumatism in three days, by the exhibition of quinine in doses of five grains, combined with the same quantity of rhubarb three times a day. Having a very severe and protracted case on hand at the time, I tried his plan four days, but without his success, and was obliged to relinquish all hopes of benefiting my patient in this way. I should think such a method applicable to those cases only which have their origin in marshy districts, and which are allied to intermittent fever.

Much has been said in favor of the tincture of colchicum in the treatment of gouty and rheumatic affections, and certainly I have found no single remedy so efficacious in relieving the severe pains, and in subduing the inflammatory symptoms in this disease. I think the best way is to combine the tincture of colchicum with equal parts of the tincture of aconite, and give a tea-spoonful of the mixture three times a day, and a tea-spoonful of laudanum at bedtime. This is an effective and satisfactory method of treatment.

I am of the opinion that the guiacum is better adapted to the chronic than to the inflammatory form of rheumatism. I use the following formula: ℞. pulv. g. guaiac ℥ ss. pulv. g. arabic, simple syrup āā. ℥ ij. aqua cinam. aqua font, ℥ iv. M. Dose one ounce thrice daily. This is the only admissible form of stimulating treatment.

The last plan of treatment I shall speak of, is the free use of nitre. A scruple of nit. potassa, and half a grain of antimony tart., givin in powder every four hours, often procures great relief, and generally speaking, is an admirable way of combatting this vexatious disease. The last case I had, I treated by giving, in the first place, fifteen grains of calomel, followed by a solution of half an ounce of nitre in a pint of lemonade, to be taken in the course of twenty-four hours with a large dose of laudanum in the evening, and sufficient of the tincture of colchicum to keep the bowels free and open. The patient was much relieved in a week, and had a good convalescence. This patient is now nearly sixty years old, and has been subject to attacks of acute rheumatism since her second year. She cannot tell exactly how many times she has had the disease. Previous to coming under my care she has had it continue seven weeks, so that she could not move herself in bed. It ought to be stated here that the first attacks of acute rheumatism are generally the most severe and protracted. In the chronic form the reverse is true. I can suggest no better plan than the treatment above mentioned, for as to curing rheumatism in a day or two, or in one afternoon, as a Homeopathist once told me he had done, altogether exceeds my skill, not to say belief. I have never been so fortunate as to find any charms in the practice of medicine.—*Ibid.*

